# Dental Status of the Bulgarian Population: The Influence of Barriers to Accessing Dental Care

**DOI:** 10.7759/cureus.74833

**Published:** 2024-11-30

**Authors:** Stanislav M Nenov, Vidin K Kirkov, Boyko K Bonev

**Affiliations:** 1 Dental Public Health, Faculty of Dental Medicine, Medical University - Sofia, Sofia, BGR; 2 Health Policy and Management, Faculty of Public Health "Prof. Tzekomir Vodenitcharov, МD, DSc" Medical University - Sofia, Sofia, BGR

**Keywords:** barriers to accessing dental services, cost of treatment, decayed missing and filled teeth (dmft) index, decayed teeth, dental fear, dental status, filled teeth, missing teeth, negative experience, prevalence

## Abstract

Introduction

Dental caries is the most common disease worldwide and affects more than 90% of Europeans. The dental status of a population is an important indicator of quality of life. Different factors act as barriers and can obstruct access to dental services. Patients experiencing such barriers have worse dental status than their counterparts.

Methods

We conducted a cross-sectional study utilizing an anonymous, self-administered questionnaire and dental checkups among 416 Bulgarians. The study design and instruments were approved by the Ethics Committee of the Medical University, Sofia, and the research was conducted in accordance with the World Medical Association Declaration of Helsinki as revised in 2013.

Results

Prevalence of dental caries by persons (E_P_) was 98.56%, and intensity (decayed, missing, and filled teeth (DMFT)) was 16.25. Prevalence per tooth (E_T_) was 52.7% for the sample, and average values of decayed (D), missing (M), and filled (F) teeth were D=3.41, M=2.89, and F=9.95, respectively. A plurality of participants (n=192, 46.15%) experienced complex influence of barriers to accessing dental services. The leading factors reported by respondents were psychosocial. Among patients with fear of dental procedures were registered the highest values of prevalence of dental caries by teeth (E_T_=55.86%), intensity (DMFT=17.21), and number of missing teeth (M=3.06). Patients with negative experiences of dental treatment had the most decayed teeth (D=4.33) within the sample. Individuals who experienced a lack of access to dental services had the most filled teeth (F=10.86). Values of E_T_, DMFT, D, M, and F were higher among patients for whom fear of dental procedures and cost of treatment were barriers than for their counterparts. Prevalence (E_T_=53.53%) and intensity (DMFT=16.5) were higher among individuals who perceived lack of pain and complaints as a barrier to accessing services.

Conclusion

Dental caries affected almost the entire Bulgarian population, which corresponded to the trend toward widespread dental diseases in Europe. Barriers to accessing dental services showed complex interactions. Psychosocial factors such as dental fear, lack of pain, and lack of time were the leading ones according to participants. Negative experience of dental treatment had the greatest impact on dental health compared to the other barriers. Patients with negative experiences had a significantly higher number of decayed teeth than their counterparts. More decayed teeth were also registered among patients for whom cost of treatment, dental fear, and lack of time were barriers. Patients with dental fear and financial obstacles had more extracted teeth than the others. Prevalence and intensity of dental caries, as well as the number of filled teeth, were relatively higher among patients who indicated dental fear, cost of treatment, lack of time, and lack of access as barriers.

## Introduction

Dental diseases are among the most common health conditions worldwide. They are not only a health problem but also pose social and economic challenges for countries. Often, dental services are only partially integrated into public health care systems or completely absent. As a result, access to appropriate and affordable dental services is a long-term goal for the majority of the world’s population. Untreated caries of permanent dentition ranks first among the 291 most common diseases [1].

The prevalence of dental caries (E_P_) among the adult population in Europe is high, reaching more than 90% [2]. That being said, the dental status of the overall European population has improved recently, with a trend towards reduction of the prevalence of caries [3]. Unfortunately, in the Republic of Bulgaria specifically, there has been a trend towards increasing spread of caries, from 93.42% in 1989 [4] to 98.29% in 2009 [5].

The intensity of dental caries (decayed, missing, and filled teeth (DMFT)) in the European region varies between 6.6 and 17.6 [2]. In Eastern European countries it varies between 12 and 19.5 [6], and for the Republic of Bulgaria it is 17.76 [5]. The number of missing teeth (M) is the main component of the DMFT index in Eastern European countries [7-9]. Many studies have shown that caries intensity (DMFT), and especially its structure, depends on social and demographic factors (i.e., age, gender, education, and frequency of visits) [4,5,9].

Access to dental care is a very important predisposing factor for utilization of services. Different factors can act as barriers and obstruct access to dental treatment. According to the Federation Dentaire International (FDI), these barriers are classified into three groups: those imposed by patients themselves, those imposed by the dental profession, and those presented by the state and society [10]. These categories correspond to the psychosocial nature of factors. Barriers by patients are described as the most important in obstructing access to services. Feelings, beliefs, and attitudes are some of the psychosocial factors that can affect a patient’s behavior regarding their dental health and pose barriers to accessing dental care. These factors interact with each other and have complex action [11]. Cost of treatment, inconvenience, fear, patient-dentist relationship, marital status, poor health, comorbidities, low education, and unrecognized health needs act as barriers to accessing dental services among elderly populations [5,10,12]. Patients often perceive dental services as too expensive and therefore choose to postpone visits to the dentist, with the cost of treatment having a stronger impact on low-income people [13-16].

Fear of the dentist and dental procedures is seen as one of the most important barriers to regular visits to a dental office [17]. Negative experiences of previous treatment contribute to the development of dental anxiety and fear of procedures [18,19]. It is believed that often the intensity of fear of the dentist outweighs the desire and need to visit a dental office. This leads to postponement and avoidance of visits by patients [5]. Assessment of treatment needs, along with dental status and age of patients, can both positively and negatively affect utilization of dental care [17]. Lack of access to services, as well as the lack of a dental office near one’s home or workplace, are factors that can obstruct access to care [13].

Barriers to accessing dental services affect the oral health of a population. The DMFT index is higher among patients who experience the negative influence of obstructing factors [20]. These patients have more decayed, missing, and filled teeth compared to their counterparts [21-23].

## Materials and methods

Objective

The aim of this study was to systematically investigate the dental status of the Bulgarian population, and especially the spread (E_T_ and E_P_) and intensity of dental caries (DMFT) among it. Moreover, there is evidence that dental status is influenced by attendance patterns of patients as well as obstructing factors that exist [20-25]. Another objective of the present study was thus to explore barriers to accessing dental services as perceived by the Bulgarian population and to analyze their impact on the dental status of patients.

Study design

To achieve the aim, we conducted a cross-sectional study comprising an anonymous self-administered questionnaire and dental checkups. The study design and main instruments (questionnaire and dental status registration card) were approved by the Ethics Committee of Medical University, Sofia (ap. No. 02/24.02.2019), and the research was conducted in accordance with the World Medical Association Declaration of Helsinki, as revised in 2013.

Participants

The cross-sectional study was conducted in dental offices in six major municipalities of the country: Sofia, Plovdiv, Burgas, Veliko Tarnovo, Vratsa, and Yambol. It included a sample of 416 patients. Participants were predominantly women (n=230, 55.29%), while men numbered 186 (44.71%). We applied a systematic random sampling technique to recruit study participants. Patients who had appointments in dental offices were asked to take part in the study; those who gave voluntary consent were included in the sample. We set an additional inclusion criterion: to be a representative of the employed part of the population (aged 18-65 years). The criterion for exclusion was age under 18 or over 65 years.

Instruments

The main instruments of the study were a self-administered questionnaire and a dental status registration card.

Questionnaire

The questionnaire consisted of 19 questions and was divided into two sections (Appendix 1). The first 11 questions captured demographic characteristics, including age, gender, place of residence, level of education, and income level (Q1-Q5), a self-assessment of health status (Q6-Q8), and dentist attendance patterns (Q9-Q11). The second section assessed barriers to accessing dental services with eight multiple-choice questions regarding general barriers (Q12) and especially barriers specific to patients themselves (Q13), i.e., fear and anxiety (Q14), lack of pain (Q15), cost of treatment (Q16), lack of access (Q17), previous negative experiences (Q18), and lack of time (Q19). We selected these specific factors because they were cited multiple times in the literature as leading barriers limiting dental visits by patients [5,10-19].

Dental Status Registration Card

We prepared a dental status registration card with boxes for each tooth, in which we recorded the respective value of variables. We used the following variables to assess dental status: D: decayed tooth (dental caries); M: missing tooth (extracted); F: filled tooth.

Data collection procedure

A pilot study was performed in September 2020 (n=50) to determine the clarity, content, and face validity of the survey. It was also used for the calibration of investigators. Subsequently, the research was conducted between November 2020 and March 2021, when final questionnaires were administered to the whole sample (n=416, response rate=100%) and the dental status of all respondents was examined in dental offices. All checkups were done following guidelines for dental examination issued by the World Health Organization [26]. Prior to completing the questionnaire, patients were informed as to the study’s purpose, methods, expected results, and possible implications for research and practice. All patients were informed that participation was anonymous and voluntary, as well as that confidentiality of data was guaranteed. Participants signed informed consent and then were asked to complete the questionnaire, after which their dental status was assessed. The researchers assigned numbers to each questionnaire and corresponding dental status registration card to guarantee the anonymity and confidentiality of the participants’ personal and health data.

Data analysis

We grouped and analyzed primary data with the statistical software program R (R Foundation for Statistical Computing, Vienna, Austria, https://www.R-project.org/). We used standard descriptive statistics to calculate values and distribution of the variables decayed (D), missing (M), and filled (F) teeth; the indices prevalence (E_T_ and E_P_) and intensity (DMFT); the distribution of barriers specific to patients themselves; and reasons for occurrence of each barrier. We compared distributions of variables in different groups categorized by the barriers to accessing dental services they identified. To prove significant associations of barriers with examined variables (D, M, F, E_T_, E_P_, DMFT), Pearson’s chi-square test of homogeneity (χ^2^) was applied. We used Tukey’s multiple comparison test to prove the significance of the difference in the distribution of variables between groups of patients. The significance level was set at p-value < 0.05.

## Results

We examined a total number of 12,778 teeth among patients in the sample (n=416). Decayed (D) accounted for 1,419 teeth (11.13%), and the mean of D for the sample was 3.41. Filled (F) were 4,137 teeth (32.3%), and the mean was F=9.95. The total number of missing teeth (M) for the sample was 1,203 (9.24%), and the mean was M=2.89. We found that the prevalence of dental caries by persons (E_P_) for the sample was 98.56%, and prevalence per teeth (E_T_) was 52.7%. The intensity of dental caries (DMFT) was thus 16.25 for the sample (Table 1).

**Table 1:** Spread of dental caries in the sample The data is presented as n (%) D: decayed teeth; M: missing teeth; F: filled teeth

Caries morbidity						
	Minimum	1^st^ Quartile	Median	Mean	3^rd^ Quartile	Maximum
D n (%)	0 (0%)	0 (0%)	2.00 (6.35%)	3.41 (11.13%)	5.00 (16.67%)	21.00 (65.62%)
M n (%)	0 (0%)	0 (0%)	2.00 (6.25%)	2.89 (9.24%)	4.00 (12.5%)	32.00 (100 %)
F n (%)	0 (0%)	5.00 (17.9%)	10.00 (31.2%)	9.95 (32.3%)	14.00 (44.8%)	29.00 (90.6%)
Prevalence by teeth (Ет) %	0%	37.5%	53.1%	52.7%	68.8%	100%

**Figure 1:**
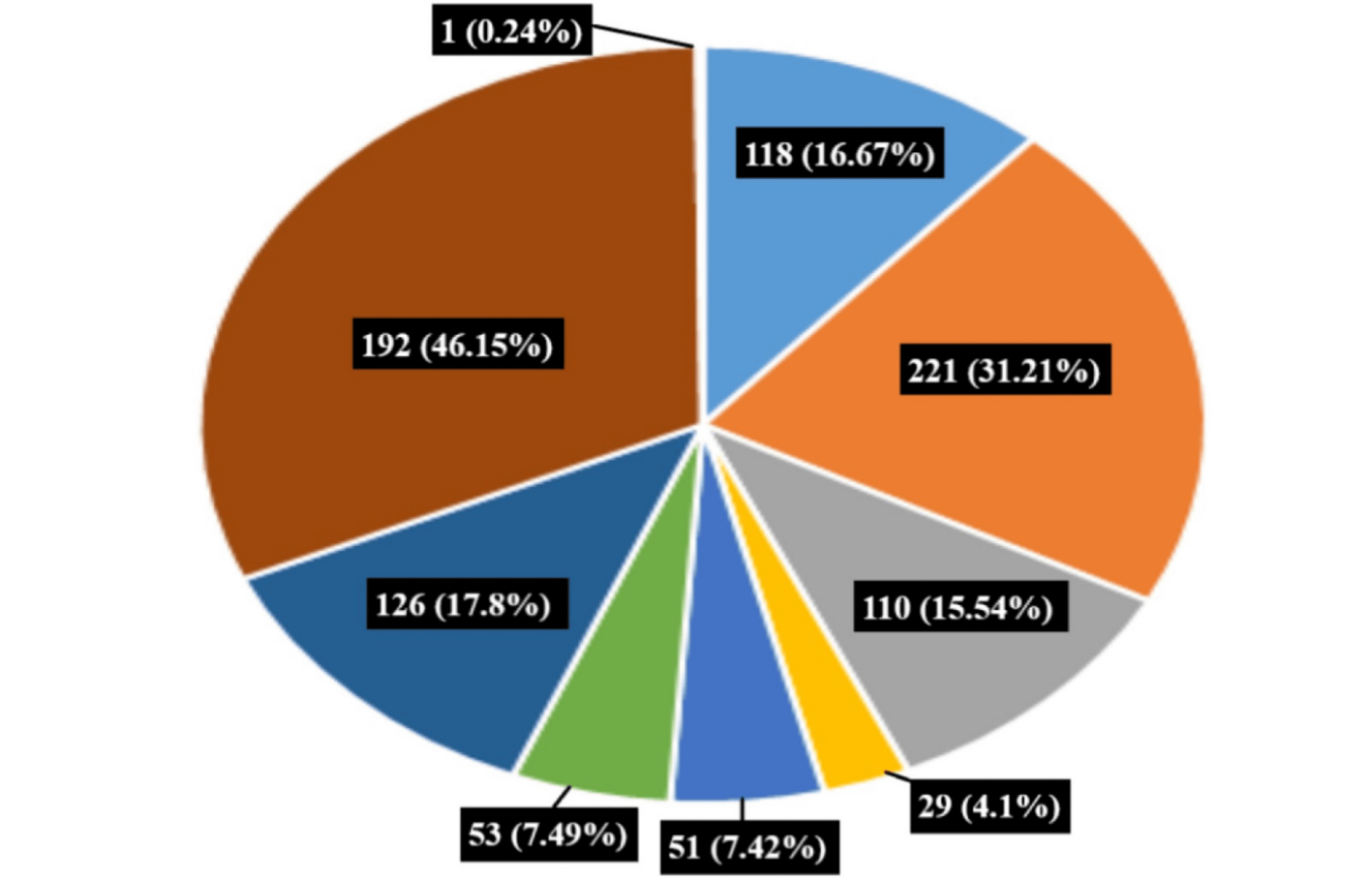
Barriers to accessing dental services (n (%)) dental fear and anxiety: 118 (16.67%); lack of pain: 221 (31.21%); cost of treatment: 110 (15.54%); lack of access: 29 (4.1%); negative experience: 51 (7.42%); comorbidities: 53 (7.49%); lack of time: 126 (17.8%); complex: 192 (46.15%); no answer: 1 (0.24%)

We asked patients about their opinion of barriers to accessing dental services. A large plurality of participants (n=192, 46.15%) experienced a complex combination of barriers. The respondents in the sample identified lack of pain as their main reason for avoiding dental appointments (n=221, 31.21%). Second in importance was lack of time (n=126, 17.8%). Lack of access to dental services had the lowest impact, according to patients (n=29, 4.1%) (Figure 1).

We asked the participants about the main reasons for the occurrence of each barrier. They indicated pain during the dental procedure as the leading reason for dental fear and anxiety (n=171, 30.54%). Negative thoughts about treatment (n=76, 13.57%) and fear of dental procedures (n=58, 10.36%) were other important reasons, according to the participants. Patients stated that they have postponed dental treatment due to the absence of any subjective complaints (n=269, 50.28%), as well as due to negligence of dental prevention (n=152, 28.41%). Patients perceived the cost of treatment as a barrier mainly due to the high price of dental procedures (n=166, 37.05%) and due to subjective factors (low income, other priorities, etc.) among 96 participants (21.43%). Patients experienced a lack of access to dental service when there was no dental office near their home or workplace (n=110, 29.73%). Access was also limited by difficulties in setting an appointment (n=82, 22.16%). Prior negative experiences cited included unsuccessful previous treatment (n=172, 41.15%) and inappropriate behavior of a dentist (n=89, 21.29%).

The results of the study showed that the prevalence of dental caries per tooth (E_T_=55.86%), intensity (DMFT=17.21), and number of missing teeth (M=3.06) were highest among patients with fear of dental procedures. Values of E_T_ and DMFT were also higher than the mean in groups for whom cost of treatment, lack of pain, and lack of access were barriers to accessing dental services. The number of missing teeth was higher than the mean in groups where barriers included cost of treatment and lack of pain. The number of decayed teeth (D=4.33) was highest among patients with prior negative experience of dental treatment. It was also higher than the mean in groups for whom dental fear, cost of treatment, and lack of time were barriers to accessing dental care. The number of filled teeth (F=10.86) was highest among individuals perceiving a lack of access to dental services. The number of filled teeth was higher than the mean in groups with dental fear, lack of pain, and comorbidities as barriers.

Prevalence (E_T_=50.04%), intensity (DMFT=15.25), number of decayed teeth (D=2.76), and number of missing teeth (M=1.7) were lowest among patients naming comorbidities as a barrier to accessing dental services. Values of E_T_ and DMFT were also lower than the mean among patients for whom negative experience and lack of time were barriers. Patients who perceived lack of pain as a barrier had fewer decayed teeth than the mean for the sample. Missing teeth were fewer than the mean for the sample in groups with barriers such as negative experience, lack of time, and lack of access. Patients naming lack of time as a barrier had the fewest filled teeth (F=9.32) of any group. The number of filled teeth was also lower than the mean for the sample in groups for which cost of treatment, negative experience, and lack of time acted as barriers (Table 2).

**Table 2:** The influence of barriers to accessing dental services on dental status of patients The data is presented as ‘Mean ± SD’ DMFT: decayed, missing, and filled teeth

	Barriers to accessing dental services by patients							
	Dental fear	Lack of pain	Cost of treatment	Lack of access	Negative experience	Comorbidities	Lack of time	Mean
Е_т_±SD	55.86%±23.82%	53.53%±23.99%	55.33%±22.66%	54.32%±23.00%	52.36%±22.37%	50.04%±20.80%	50.49%±23.05%	52.40%±23.27%
D±SD	3.93±4.52	3.3±4.09	4.1±4.15	3.41±3.31	4.33±5.32	2.76±3.51	3.71±4.34	3.4±4.22
F±SD	10.22±6.10	10.31±6.19	9.99±5.87	10.86±5.51	9.33±5.83	10.79±4.79	9.32±5.62	10.08±6.09
M±SD	3.06±4.58	2.89±4.51	2.95±4.04	2.45±3.48	2.41±4.09	1.7±2.73	2.55±3.58	2.65±4.05
DMFT±SD	17.21±7.53	16.5±7.56	17.04±7.23	16.72±7.15	16.08±7.07	15.25±6.42	15.57±7.25	16.13±7.3

We compared values of E_T_, DMFT, D, M, and F between groups experiencing and not experiencing the same barrier. These indicators were higher in groups with barriers such as fear of dental procedures and cost of treatment than in groups without these barriers. Prevalence per tooth (E_T_), intensity (DMFT), and number of filled teeth (F) were higher among patients for whom lack of pain and complaints was a barrier than for their counterparts. The same result was registered among patients for whom lack of access was a barrier. Group with prior negative experiences of dental care and group that indicated lack of time as a barrier presented with more decayed teeth (D) but fewer filled teeth (F) than groups without such barriers. Patients who identified comorbidities as a barrier had more filled teeth (F) than others. Analysis of variance proved an association between the variables decayed teeth (D) and cost of treatment, as well as between the variables missing teeth (M) and comorbidities (Pearson’s chi-square, p-value < 0.05) (Table 3).

**Table 3:** The impact of barriers to accessing dental care on dental status The data is presented as ‘Mean ± SD’ Pearson’s chi-square test of homogeneity (χ^2^) was used and the significance level was set at p-value<0.05. DMFT: decayed, missing, and filled teeth

Barriers to accessing dental services				
	Dental fear and anxiety		χ^2^ (Pearson’s chi-square test of homogeneity)	
	Yes (n=118)	No (n=297)	χ^2^	p-value
Ет±SD	55.86%±23.82%	51.64%±22.49%	2.88	0.091
D±SD	3.93±4.52	3.22±3.86	2.63	0.11
F±SD	10.22±6.1	9.87±5.81	0.3	0.58
M±SD	3.06±4.58	2.84±4.34	0.22	0.64
DMFT±SD	17.21±7.53	15.92±7.07		
	Lack of pain		χ^2^ (Pearson’s chi-square test of homogeneity)	
	Yes (n=221)	No (n=194)	χ^2^	p-value
Ет±SD	53.53%±23.99%	52.05%±21.69%	0.43	0.51
D±SD	3.3±4.09	3.55±4.05	0.38	0.54
F±SD	10.31±6.19	9.58±5.52	1.57	0.21
M±SD	2.89±4.51	2.91±4.28	0	0.97
DMFT±SD	16.5±7.56	16.04±6.83		
	Cost of treatment		χ^2^ (Pearson’s chi-square test of homogeneity)	
	Yes (n=110)	No (n=305)	χ^2^	p-value
Ет±SD	55.33%±22.66%	51.94%±22.99%	1.77	0.18
D±SD	4.1±4.15	3.17±4.01	4.23	0.04
F±SD	9.99±5.87	9.96±5.9	0	0.96
M±SD	2.95±4.04	2.88±4.53	0.02	0.9
DMFT±SD	17.04±7.23	16.02±7.21		
	Lack of access		χ^2 ^(Pearson’s chi-square test of homogeneity)	
	Yes (n=29)	No (n=386)	χ^2^	p-value
Ет±SD	54.32%±23.00%	52.73%±22.95%	0.13	0.72
D±S D	3.41±3.31	3.42±4.12	0	0.99
F± SD	10.86±5.51	9.9±5.92	0.72	0.4
M±SD	2.45±3.48	2.93±4.47	0.33	0.57
DMFT±SD	16.72 ± 7.15	16.25 ± 7.23		
	Negative experience		χ^2^ (Pearson’s chi-square test of homogeneity)	
	Yes (n=51)	No (n=364)	χ^2^	p-value
Ет±SD	52.36%±22.37%	52.90%±23.03%	0.03	0.87
D±SD	4.33±5.32	3.29±3.85	2.95	0.087
F±SD	9.33±5.83	10.06±5.9	0.68	0.41
M±SD	2.41±4.09	2.97±4.45	0.71	0.4
DMFT±SD	16.08±7.07	16.32±7.25		
	Comorbidities		χ^2^ (Pearson’s chi-square test of homogeneity)	
	Yes (n=53)	No (n=362)	χ^2^	p-value
Ет±SD	50.04%±20.8%	53.25%±23.22%	0.9	0.34
D±SD	2.76±3.51	3.52±4.14	1.62	0.2
F±SD	10.79±4.79	9.85±6.03	1.19	0.28
M±SD	1.7±2.73	3.08±4.57	4.56	0.033
DMFT±SD	15.25±6.42	16.44±7.33		
	Lack of time		χ^2^ (Pearson’s chi-square test of homogeneity)	
	Yes (n=126)	No (n=289)	χ^2^	p-value
Ет±SD	50.49%±23.05%	53.86%±22.83%	1.91	0.17
D±SD	3.71±4.34	3.29±3.94	0.9	0.34
F±SD	9.32±5.62	10.25±5.99	2.22	0.14
M±SD	2.55±3.58	3.05±4.72	1.15	0.28
DMFT±SD	15.57±7.25	16.6±7.2		

Patients were categorized according to whether they had experienced a single major barrier to accessing dental services or multiple barriers; this served as a starting point for investigation of whether their dental status depended on the existing barriers. We found that values of prevalence per tooth (E_T_=61.57%), intensity (DMFT=18), and number of decayed teeth (D=11.33) were highest in the group with prior negative experiences of dentistry; the number of filled teeth (F=6.67), however, was lowest in the same group. The highest average number of filled teeth (F=12.17) occurred in the group with lack of access, while the number of missing teeth (M=4.06) was highest in the group with dental fear and anxiety. Values of E_T_ (45.17%) and DMFT (13.9) were lowest in the group indicating only lack of time as a barrier, while the lowest number of decayed teeth (D=2.51) was found in groups for whom lack of pain and lack of access were barriers. Statistical analysis proved an association between the variables decayed teeth and barriers to accessing dental services (Pearson’s chi-square, p-value < 0.05) (Table 4).

**Table 4:** Association between dental status and barriers to accessing dental services The data is presented as ‘Mean ± SD’ Pearson’s chi-square test of homogeneity (χ^2^) was used and the significance level was set at p-value<0.05. DMFT: decayed, missing, and filled teeth

	Barriers to accessing dental services by patients								χ^2^ (Pearson’s chi–square test of homogeneity)	
	Dental fear	Lack of pain	Cost of treatment	Lack of access	Negative experience	Comorbidities	Lack of time	Multiple barriers	χ^2^	P value
Ет±SD	52.74%±22.38%	54.16%±23.56%	52.95%±20.11%	54.87%±15.44%	61.57%±19.28%	48.07%±27.05%	45.17%±21.43%	54.27%±23.34%	1.08	0.38
D±SD	3.39±3.98	2.51±3.38	3.31±3.61	2.51±1.38	11.33±6.66	2.67±3.06	2.98±3.59	3.94±4.45	3.01	0.0043
F±SD	8.86±5.38	10.73±6.41	9.42±5.12	12.17±5.57	6.67±4.04	10.17±6.94	7.84±4.75	10.4±5.95	1.78	0.09
M±SD	4.06±6.39	3.55±5.47	3.85±4.38	2.5±2.35	0±0	1.92±1.93	3.08±4.04	2.32±3.51	1.6	0.14
DMFT±SD	16.31±7.07	16.78±7.36	16.58±6.54	17.17±5.23	18±4.58	14.75±8.19	13.9±6.57	16.66±7.42		

Analysis of the variance with multiple comparisons of distributions of number of decayed teeth (D) between the group with negative experience and other groups proved the significance of registered differences in the values of D (Tukey, p adjusted<0.05), as shown in Table 5.

**Table 5:** Multiple comparisons of values of decayed teeth (D) between group with negative experience and other groups Statistical test: Tukey multiple comparisons of means (95% family-wise confidence level) The significance level was set at p-value (p adjusted)<0.05.

Index	Leading barrier to accessing dental care by patients		Diff	P adjusted
D	Negative experience	Multiple	7.395833	0.0339
	Negative experience	Cost of treatment	8.025641	0.0239
	Negative experience	Dental fear	7.944444	0.0228
	Negative experience	Lack of access	8.833333	0.0399
	Negative experience	Lack of pain	8.827839	0.0047
	Negative experience	Lack of time	8.353741	0.0115
	Negative experience	Comorbidities	8.666667	0.0193

## Discussion

The current study aimed to investigate dental status among the Bulgarian population and how it was influenced by barriers to accessing dental services. The results of the study showed that the prevalence of dental caries by persons (E_P_) in the Bulgarian population was 98.56%, which corresponded to the trend for the widespread nature of dental caries among the European population [2]. However, comparison to previous studies showed that E_P_ had increased over time from 93.42% in 1989 [4] and 98.29% in 2009 [5]. We found that the prevalence of caries per tooth (E_T_) in the Bulgarian population was 52.7%, which corresponded to the global trend [27].

Results of the study showed that DMFT in the Bulgarian population was 16.25, a reduction from 2009, when it was 17.76 [5]. This corresponded to values of DMFT in Eastern Europe, where it varies between 12 and 19.5 [3,6]. Contrary to the results in other countries [7-9], where the number of missing teeth (M) was the main component of the DMFT index, in the Republic of Bulgaria the main component of DMFT was the number of filled teeth (F=9.95). The results of the study showed an increase in the number of decayed teeth (D) from 1.96 in 2009 [5] to 3.4 at the time of the study.

The results of the study showed that a near-majority of participants (46.15%) were influenced by the complex interaction of multiple barriers to accessing dental care. The main reason for not visiting a dentist was lack of pain, which corresponded to previous studies [13]. The current study indicated that cost of treatment was a less important barrier, which was contrary to considerations by other authors [12,14-16]. Dental fear had a sizeable influence on utilization of dental services in Bulgaria; this corresponds with the results of previous studies [17]. Negative experience of previous treatment was another important barrier, which also corresponded to results by Kyle and Singh [18,19].

Prevalence (E_T_=53.53%) and intensity (DMFT=16.5) were higher among individuals who identified lack of pain and complaints as a barrier to accessing services. This result tends to confirm conclusions by Crocombe et al. [20]. Prevalence per tooth (E_T_), intensity (DMFT), and numbers of decayed (D), missing (M), and filled teeth (F) were higher among patients who experienced dental fear than among their counterparts. These results corresponded to the results of some previous studies [21-23] while contradicting others [28-30].

Patients who indicated cost of treatment as a barrier had more decayed teeth (D=4.1) than their counterparts (D=3.17), which corresponded to the results by Thompson et al. [24]. Decayed teeth (D=4.33) were more common among patients who reported prior negative experiences as a major barrier than in other groups; these results were in line with findings by Flink et al. [25].

This study provides valuable results on the dental status of the Bulgarian population and its association with barriers to accessing dental services; however, it has some limitations. First, the method used for gathering sociological data (i.e., the questionnaire survey) always leaves doubt as to whether the respondents answered honestly. In addition, the participation of multiple investigators in dental examinations of patients always increases the risk of registration errors. In light of these considerations, the current outcomes should be interpreted with caution. Second, the cross-sectional design of the study provides only information about the current situation; future researchers should consider a longitudinal design to study the dynamics of these phenomena over time. Third, the limited sample size (n=416) might have affected the comprehensiveness of the results and may hinder the generalizability of the data. In this aspect, future research should attempt to increase the number of patients included in the sample. Finally, this study reported only national data; future investigations in other countries would allow for comparisons on a multinational level.

Despite these limitations, the current study provides useful information on the influence of barriers to accessing dental services on the dental status of a population. This impact should be considered seriously by both dentists and patients, as it directly affects dental health and quality of life for the population. Improvement of access to services is expected to result in better oral health status of patients.

## Conclusions

Dental diseases affected almost the entire Bulgarian population (E_P_=98.56%), and the intensity of dental caries (DMFT) was 16.25. These results corresponded to data for European countries and the trend toward widespread dental caries among Europeans. Contrary to the results in other countries, where the number of missing teeth (M) was the main component of the DMFT index, in Bulgaria the main component was the number of filled teeth (F).

Barriers to accessing dental services influenced utilization of dental care. Negative experiences of dental treatment had the greatest impact on dental status; patients with negative experiences had significantly more decayed teeth than their counterparts. Patients for whom cost of treatment, dental fear, and lack of time were barriers had significantly more decayed teeth than the others. Patients with dental fear and financial obstacles had more extracted teeth than the others. Prevalence and intensity of dental caries, along with the number of filled teeth, were relatively high among patients who indicated dental fear, cost of treatment, lack of time, and lack of access as barriers.

## Appendices

Appendix 1 

**Table 6:** Questionnaire

	Part 1	Demographic characteristics								
№	Question	Answers								
		A	B	C	D	E	F	G	H	I
1	Your gender is:	Male	Female							
2	Your age is:	18–29 years	30–44 years	45–65 years						
3	Your place of residence is:	Urban area	Rural area							
4	Your education is:	Without	Primary school	Secondary school	College	University				
5	Your income is:	Without	< 610	610–1000	1000–1500	1500–2000	2000–2500	2500–3000	> 3000	
6	Do you have any comorbidities? (multiple-choice)	No	Cardiovascular	Respiratory	Hepatic	Endocrine	Musculoskeletal	Other		
7	How do you assess your general health?	Excellent	Good	Satisfactory	Bad					
8	How do you assess your dental health?	Excellent	Good	Satisfactory	Bad					
9	When was your last visit to dental office?	A month ago	Six months ago	One year ago	Two years ago	More than two years ago				
10	What was the reason of your last visit?	Checkup	Pain	Treatment						
11	You visit dental office for checkup:	Every six months	Annually	Several years	On emergency					
	Part 2	Barriers to accessing dental services								
№	Question	Answers								
	(multiple-choice)	A	B	C	D	E	F	G	H	I
12	Which is the main group of barriers to accessing dental health?	By patients	By dental profession	By state and society						
13	Which are main barriers by patients themselves?	Fear and anxiety	Lack of pain	Cost of treatment	Lack of access	Negative experience	Comorbidities	Lack of time		
14	Your dental fear is caused by:	Pain during the dental procedure	Dental treatment	Dental equipment	Dental anesthesia	Improper (rude) behavior of the dentist	Bad experience of previous visit	Unreasonable fear	Unpleasant thoughts about the treatment	Other reasons (please specify)
15	If you miss or postpone dental treatment it’s due to:	Lack of knowledge about the dental health	Underestimating dental prevention	Lack of complaints	Self-treatment	Other reasons (please specify)				
16	Cost of treatment is barrier for you due to:	High price of dental treatment	Copayments	Lack of health insurance	Additional cost (transport, etc.)	Subjective factors (income, other priorities, etc.)	Other reasons (please specify)			
17	If you experience lack of access the reason/s is/are:	Remoteness of dental practice from home or workplace	Problems in communication	Difficulties in making an appointment	Inaccessible dental office (no ramps, elevator, etc.)	Discrimination	Other reasons (please specify)			
18	If you have negative experience of dental treatment, it is due to:	Previous unsuccessful treatment	Shared experience by relatives	Negative media information	Inappropriate behavior of the dentist	Other reasons (please specify)				
19	If lack of time is the reason you postpone dentist visits, is it because of:	Your work schedule	Family reasons	Other priorities	Other reasons (please specify)					
